# Sixth Annual Report of the Managers of the State Lunatic Asylum, Made to the Legislature (of New York,) February 1st. 1849

**Published:** 1849

**Authors:** 


### ARTICLE VI.

*Sixth Annual Report of the Managers of the State Lunatic Asylum, made to the Legislature (of New York,) February 1st.* 1849. (From Dr. Brigham.)

*Report of the Pennsylvania Hospital for the Insane for the year* 1848. By THOMAS S. KIRKBRIDE, M. D., Physician to the Institution. Published by order of the Board of Managers. Philadelphia, 1849. (From the author.)

*Report of the Eastern Asylum in the City of Williamsburgh, Vtrginia*, 1848. Richmond: Printed by Shepherd and Colin. 1849. (From Dr. Galt.)

There is no member of the medical profession who deserves more thanks from the community at large, and gratitude from the afflicted, for his efforts in their behalf, than the physician who, for their good, submits to the confinement of a Lunatic Asylum. Here he is continually surrounded with the most ungovernable of all patients, and liable, at all times, to become the victim of some insane impulse. His ingenuity is taxed to the utmost to devise means of controlling this patient to prevent him from doing violence to himself—that, to guard him from homicidal impulses; or another from disturbing the whole househould with his songs, his sermons, or his curses. Nor are his powers of governing the insane alone called into requisition, for he must necessarily have control of large numbers of subordinate officers, attendants, and servants who must all have their places and be required to fill them. Here he will continually meet with specimens of perversity to vex and harass him, and unless he be endowed with patience and an unusual amount of self control and equanimity of temper, he will often be thrown off his guard, lose his influence, have his disposition soured, and his happiness perverted. Nor is his prowess in government the only quality necessary in his disposition. He must, while governing, know how to submit to control. And happy will he be if, upon his board of managers there be found none but those who are willing to second all his laudable efforts to do good; nor those who will, with a limited knowledge presume to give directions contrary to his wishes which he knows will result in evil.

And again, the diseases of the brain and nervous system, are the most obscure and their manifestations or signs the most erratic of any other class of diseases; consequently requiring the closest observation and strongest powers of thought correctly to apply the means of relief. Although the salaries of these officers are generally liberal, certainly no other motive than a sense of duty could induce a physician of proper qualifications to enter upon a post of such difficulties as those we have described.

The labors of Superintendents of Hospitals for the Insane have already done much for the relief of the lunatics in our own country. The prospects, however, are still brightening. We see, almost daily, improvements being suggested and introduced, the most important of which, of late, relate to the construction of suitable buildings, and especially to heating and ventilating them.

Among the most encouraging signs of improvement is the organization of the "Association of Superintendents of American Institutions for the Insane," which is to hold its next meeting on the 21st May, at Utica, New York, the site of the Institution from which the first report, at the head of this article was issued.

This is the most extensive establishment of the kind in the United States, having room for about 500 patients and the suit of officers, attendants, &c., necessary for their care and treatment. There were, at the date of this report, 495 patients in the establishment—241 men and 254 women. In the course of the year, 877 patients were in the asylum. Besides the five resident officers of the institution, there are 88 persons in its employ.

There were discharged during the year 174 patients cured, 84improved, 38 unimproved, and 86 died.

Dysentery prevailed in the asylum and in several towns in its vicinity, extensively, during the months of August and September, having affected 240 of the patients and proving fatal to 36. This will account for the large mortality as stated above. In other respects it has been very prosperous.

The remarks, in this document, of Dr. Brigham, the distinguished superintendent of the institution, on the increase of insanity, its causes, prevention, prognosis, medical and moral treatment, are highly judicious and interesting.

The second report at the head of our article presents many encouraging features.

There were in the Hospital at the date of the last report, 188 patients. There have been admitted, during the year, 215, making 403 in the institution during the year, of whom 203 have been discharged, leaving, at the date of this report, 200 in the Hospital. Those discharged were conditioned as follows:—cured 120; much improved, 23; improved, 24; stationery, 19, and 19 died.

The author of the report, who is the superintendent of the institution, is highly pleased with the plan of separate or detached cottages, which they have adopted for such patients as are suited for their occupation by wealth and disposition.

The last of these documents shows that at the commencement of the yaar, 164 patients were in the institution, 34 have been received during the year, 16 have been discharged, and 17 died.

Dr. Galt, authorof the report and superintendent of the institution, gives a brief account of the condition of the household and takes up the consideration of the plans adopted for new buildings about to be erected for the accommodation of the convalescent and demented.

In speaking of the propriety of a detached building for those of the latter class, he says the objection on account of the liability of their being neglected, does not hold as strongly in the south as north, because the attendants have slaves to do the menial service connected with the care of patients. All classes of patients are admitted and treated in this institutionincluding slaves and free persons of color. E.

